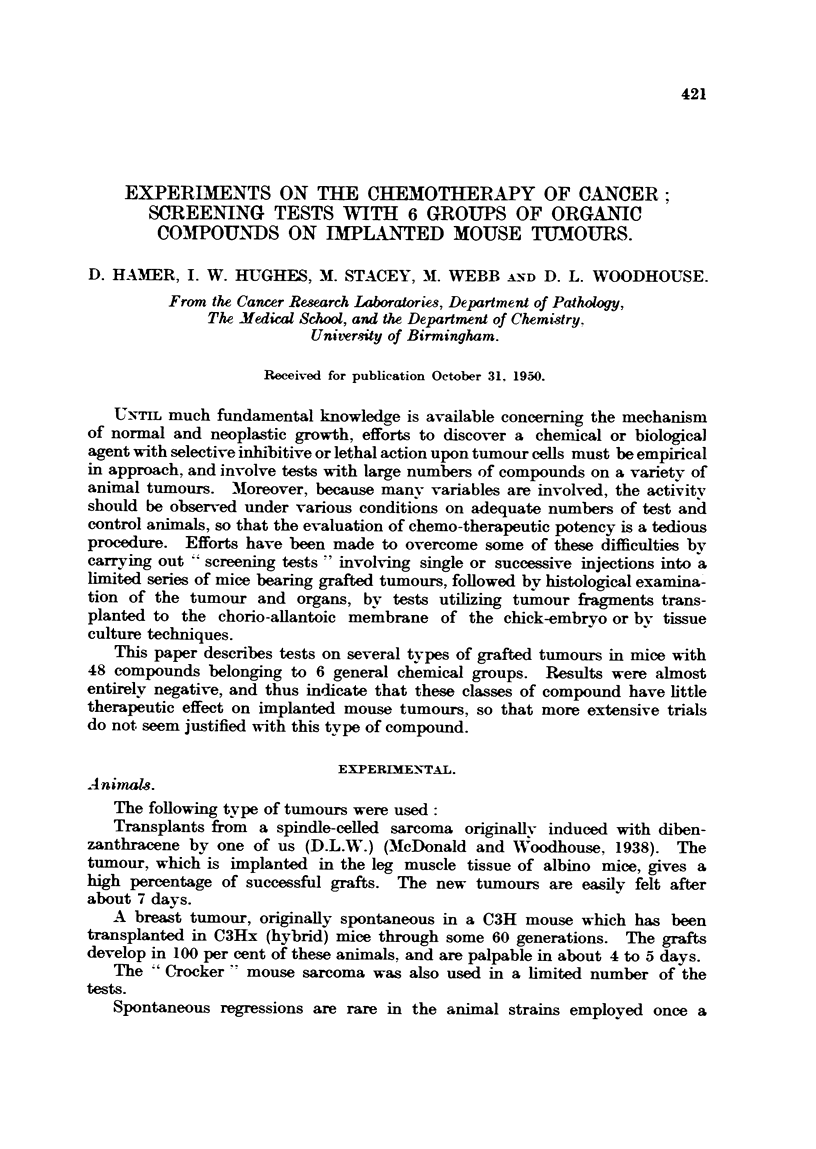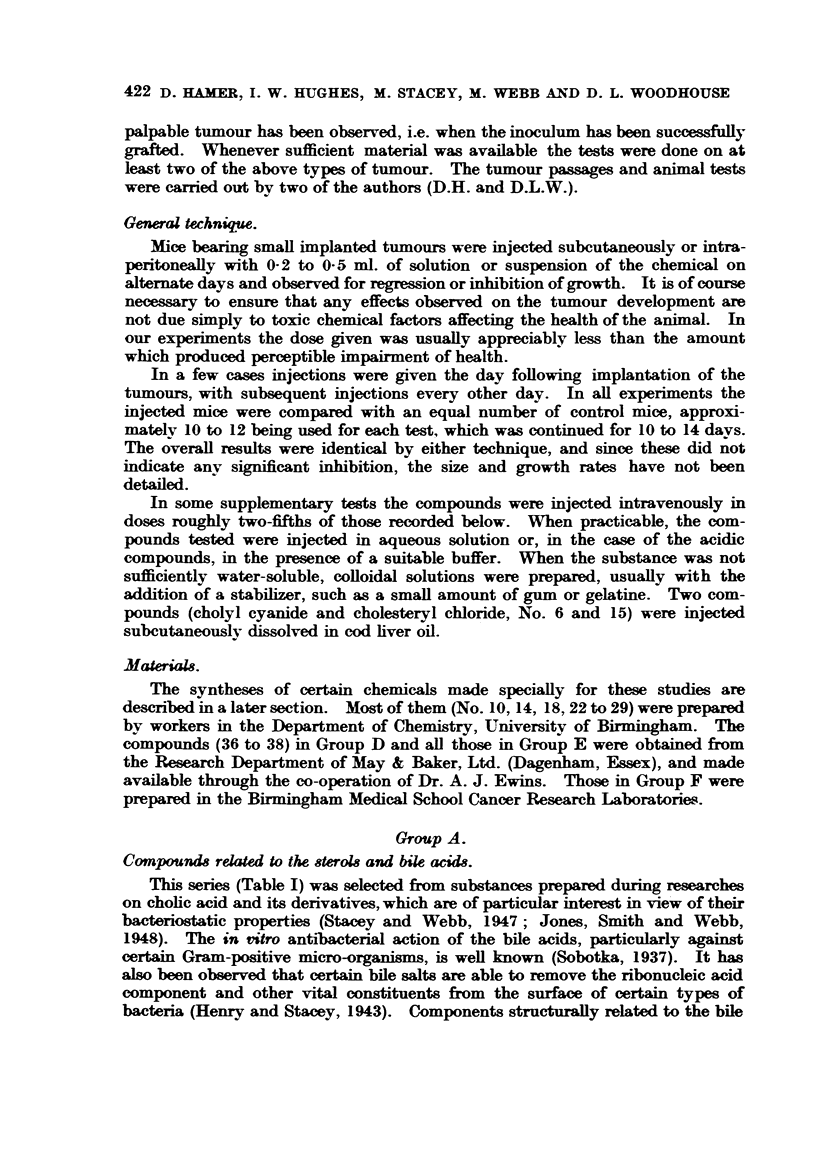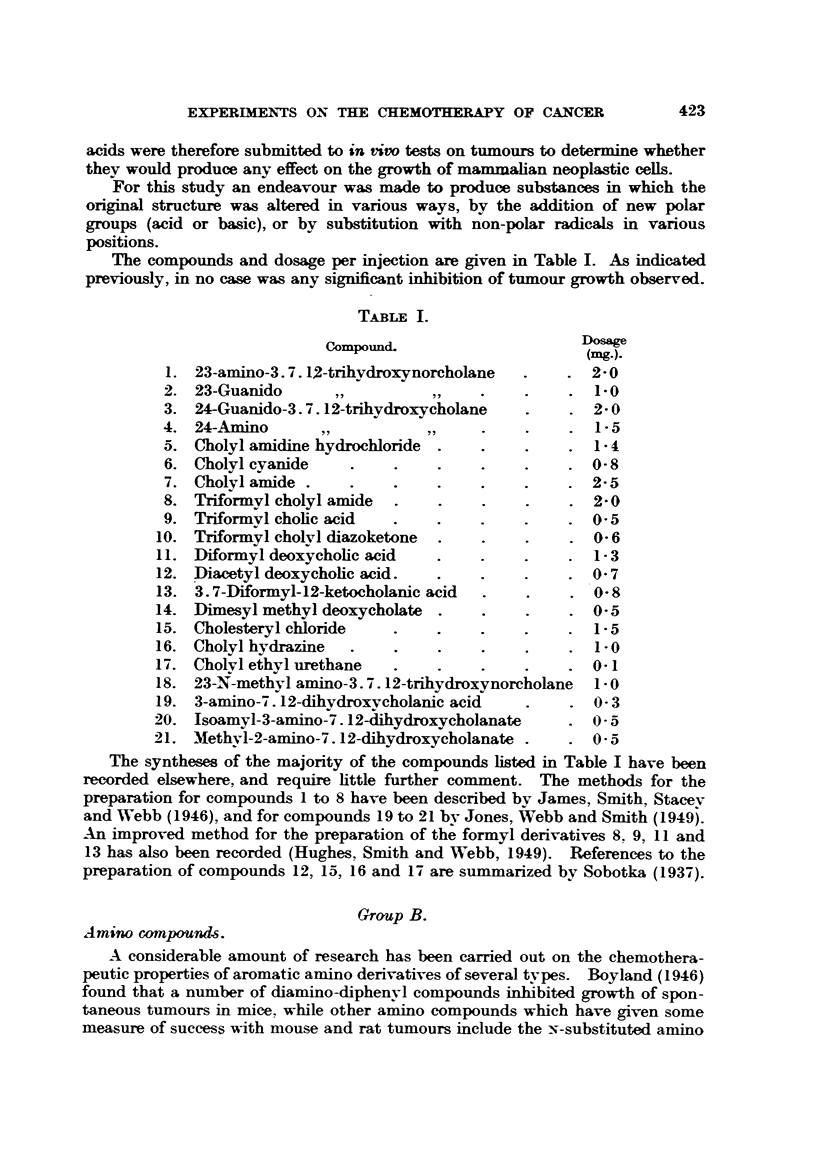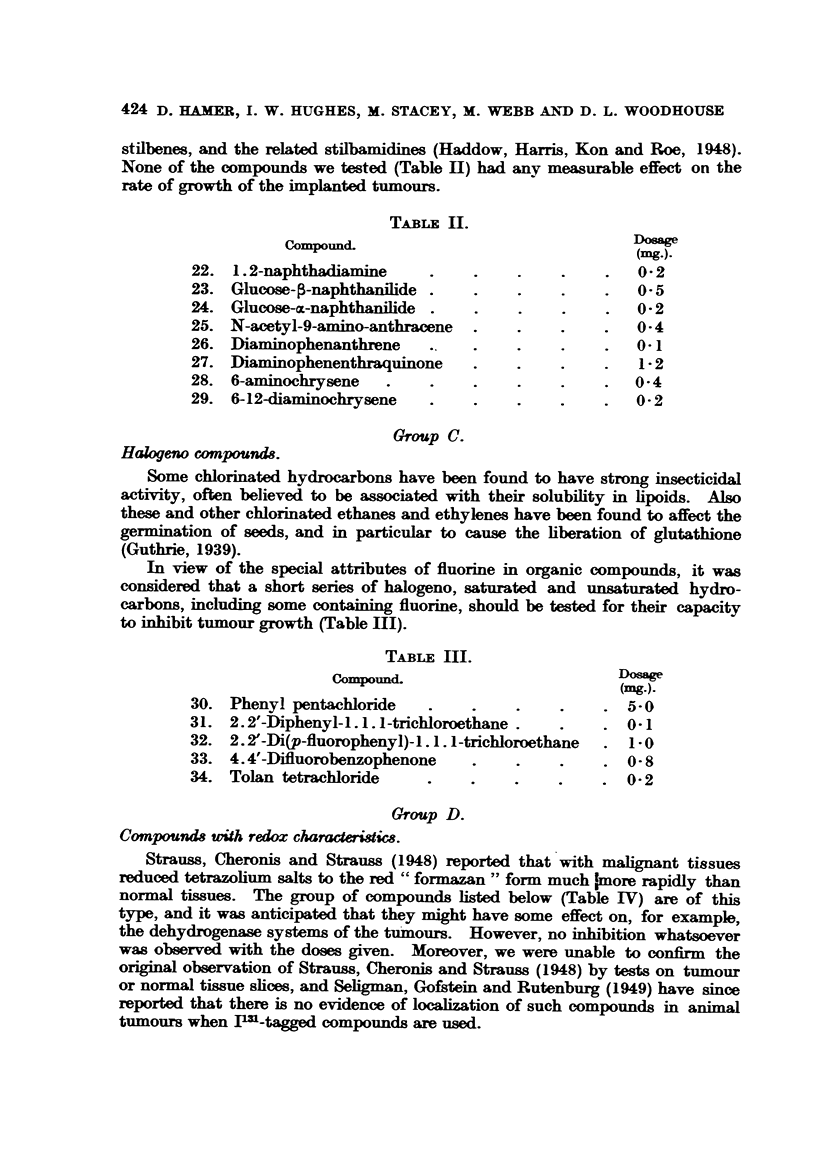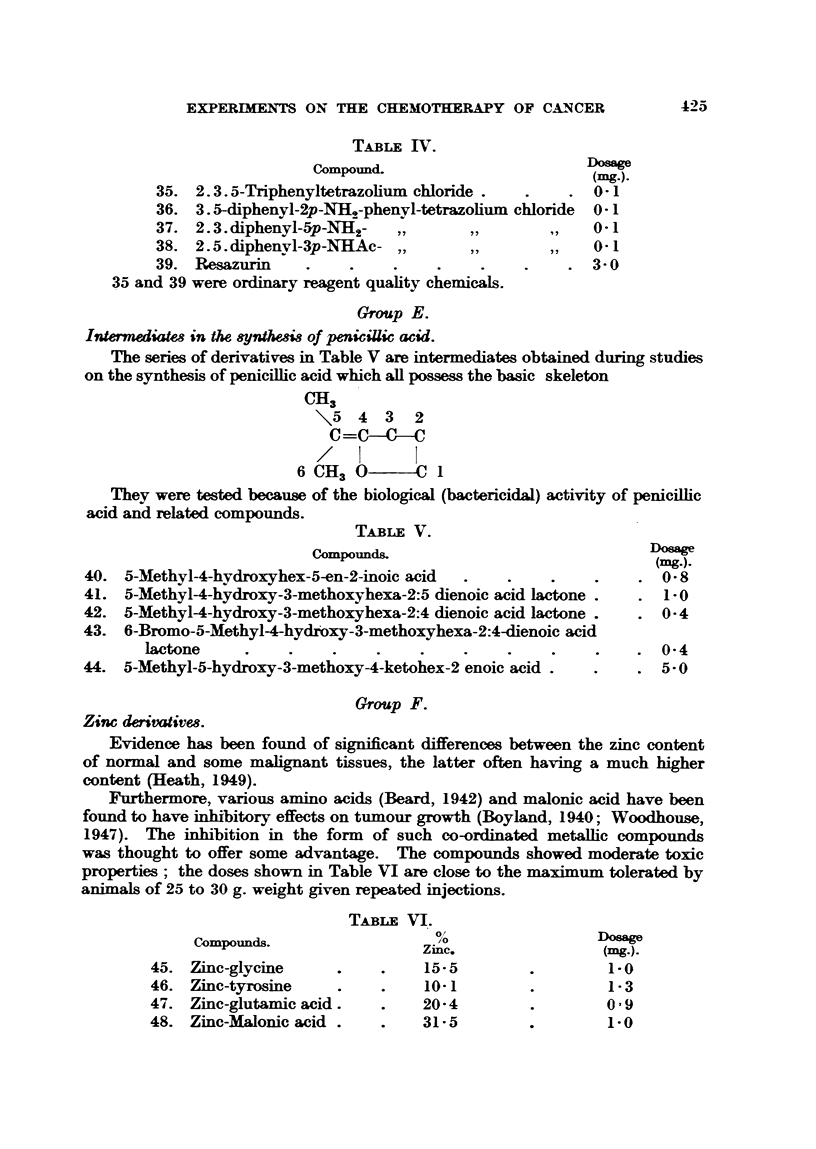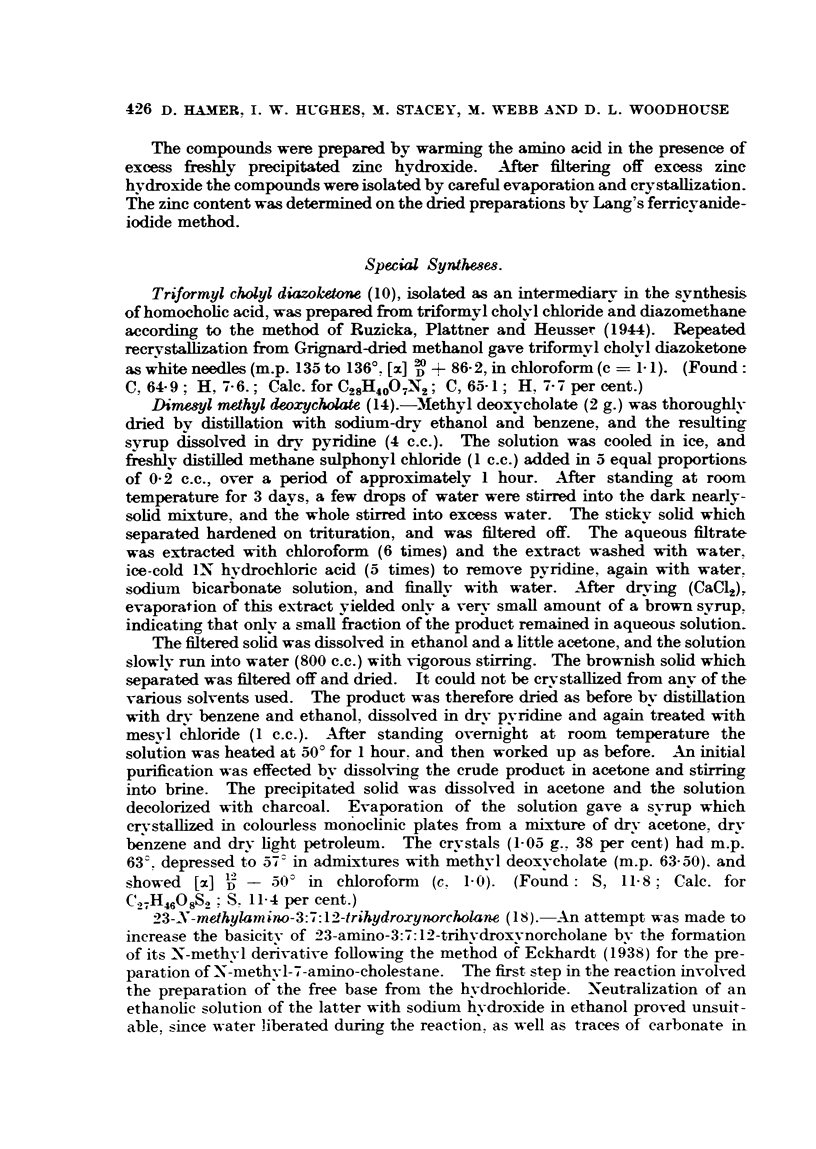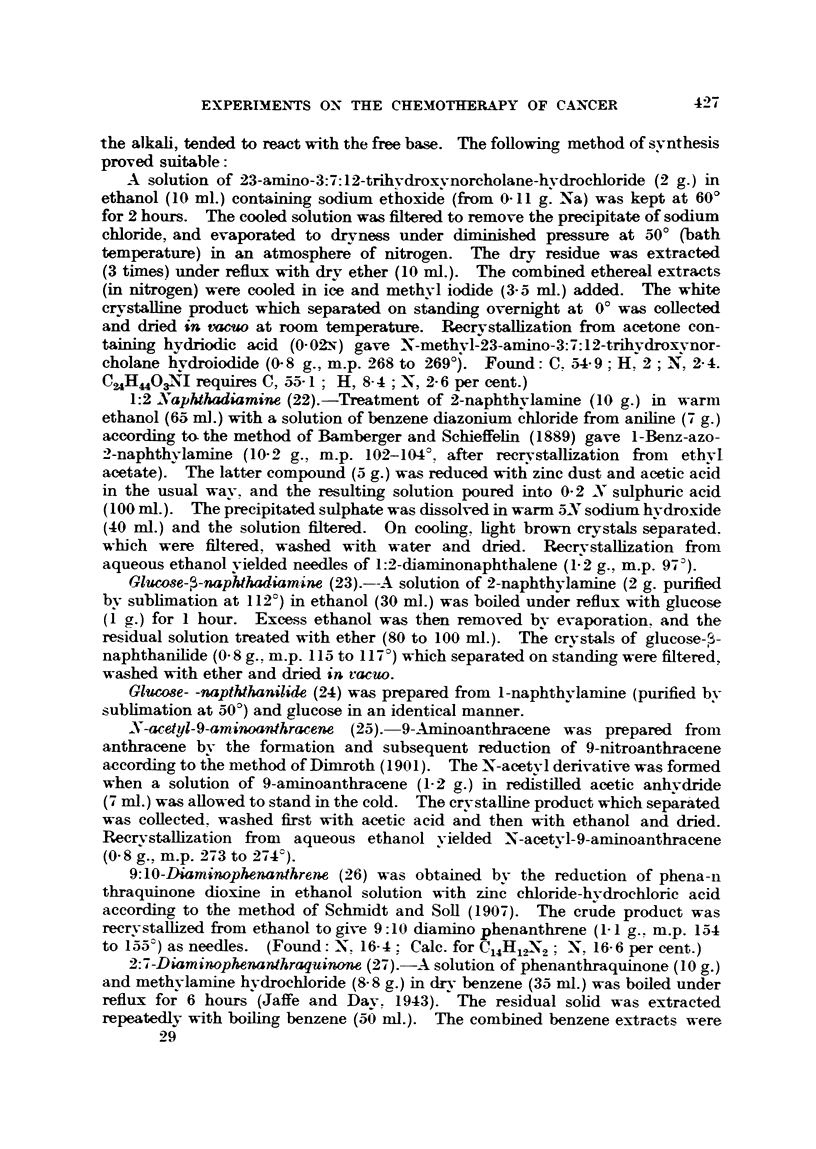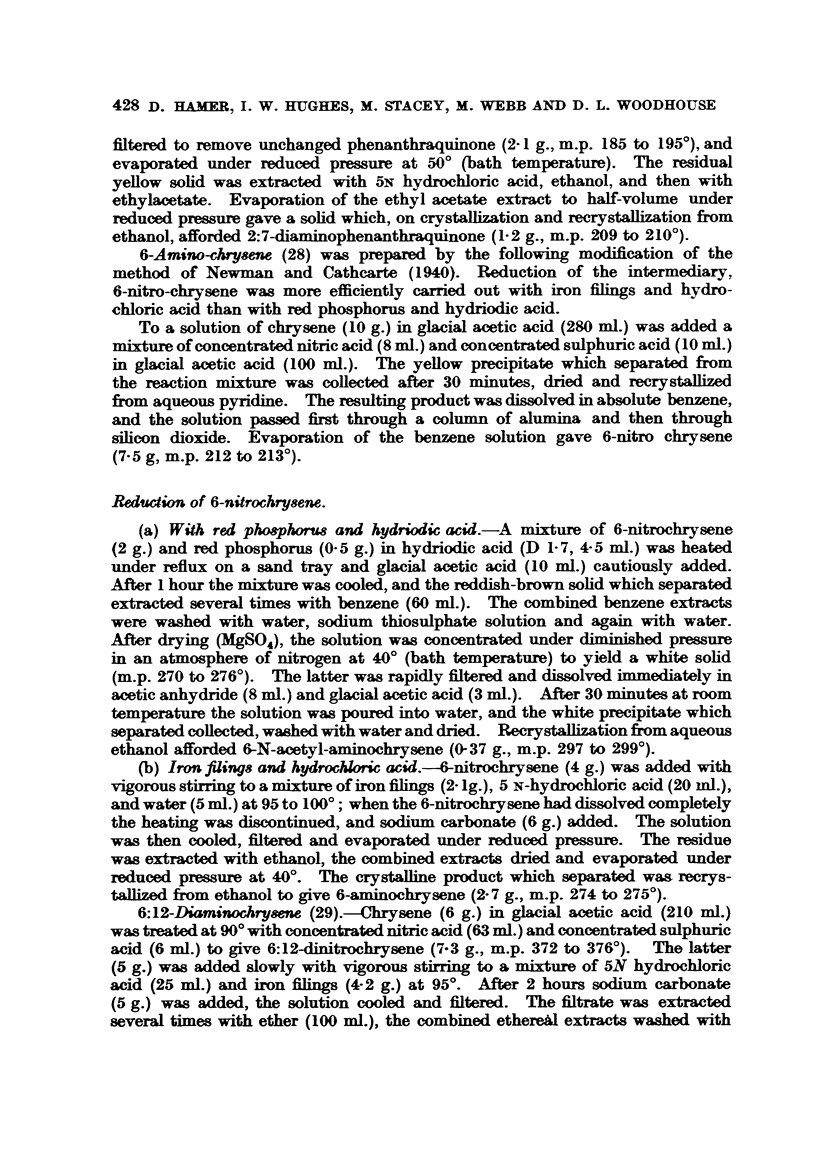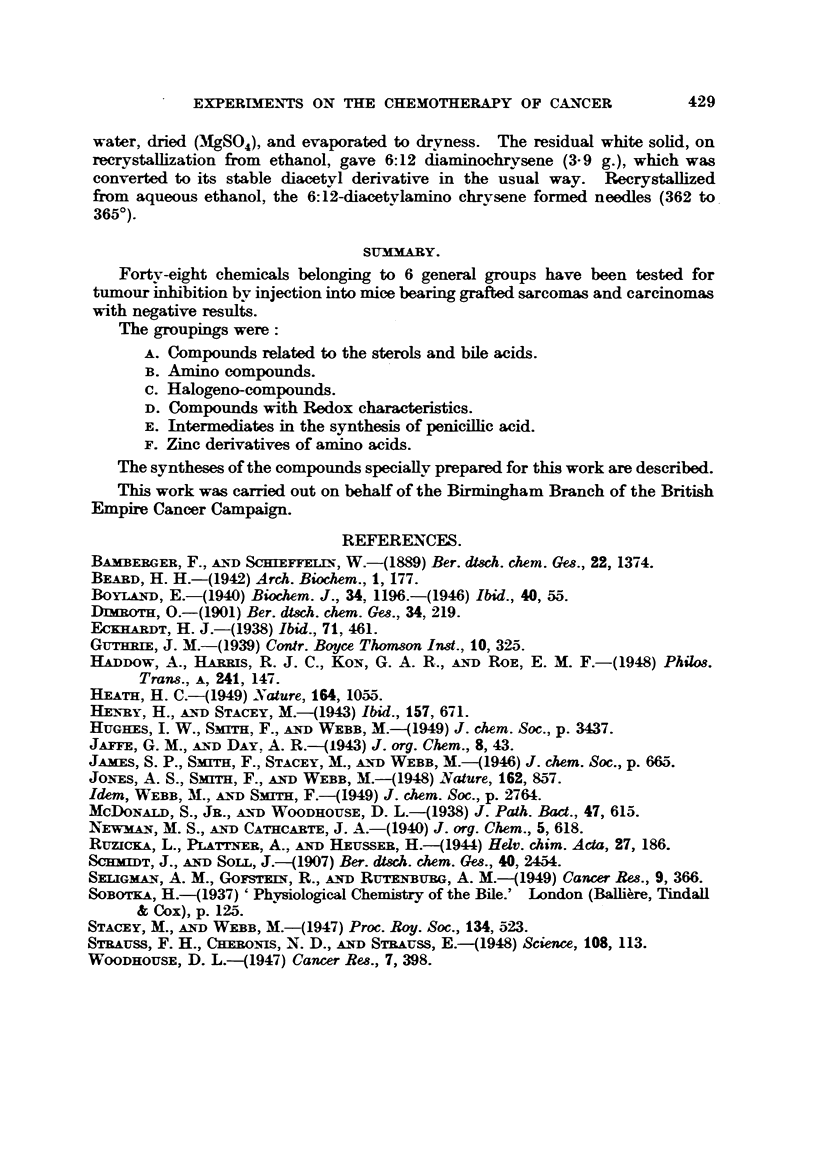# Experiments on the Chemotherapy of Cancer; Screening Tests with 6 Groups of Organic Compounds on Implanted Mouse Tumours

**DOI:** 10.1038/bjc.1950.41

**Published:** 1950-12

**Authors:** D. Hamer, I. W. Hughes, M. Stacey, M. Webb, D. L. Woodhouse


### EXPERIMENTS ON THE CHEMOTHERAPY OF CANCER; SCREENING TESTS WITH 6 GROUPS OF ORGANIC COMPOUNDS ON IMPLANTED MOUSE TUMOURS.

D. HAMER, I. W. HUGHES, M. STACEY, M. WEBB AND D. L. WOODHOUSE.

*From the Cancer Research Laboratories, Department of Pathology, The Medical School, and the Department of Chemistry, University of Birmingham.*

Received for publication October 31, 1950.

UNTIL much fundamental knowledge is available concerning the mechanism of normal and neoplastic growth, efforts to discover a chemical or biological agent with selective inhibitive or lethal action upon tumour cells must be empirical in approach, and involve tests with large numbers of compounds on a variety of animal tumours. Moreover, because many variables are involved, the activity should be observed under various conditions on adequate numbers of test and control animals, so that the evaluation of chemo-therapeutic potency is a tedious procedure. Efforts have been made to overcome some of these difficulties by carrying out " screening tests " involving single or successive injections into a limited series of mice bearing grafted tumours, followed by histological examination of the tumour and organs, by tests utilizing tumour fragments transplanted to the chorio-allantoic membrane of the chick-embryo or by tissue culture techniques.

This paper describes tests on several types of grafted tumours in mice with 48 compounds belonging to 6 general chemical groups. Results were almost entirely negative, and thus indicate that these classes of compound have little therapeutic effect on implanted mouse tumours, so that more extensive trials do not seem justified with this type of compound.

### EXPERIMENTAL.

### *Animals.*

The following type of tumours were used:

Transplants from a spindle-celled sarcoma originally induced with dibenzanthracene by one of us (D.L.W.) (McDonald and Woodhouse, 1938). The tumour, which is implanted in the leg muscle tissue of albino mice, gives a high percentage of successful grafts. The new tumours are easily felt after about 7 days.

A breast tumour, originally spontaneous in a C3H mouse which has been transplanted in C3Hx (hybrid) mice through some 60 generations. The grafts develop in 100 per cent of these animals, and are palpable in about 4 to 5 days.

The " Crocker " mouse sarcoma was also used in a limited number of the tests.

Spontaneous regressions are rare in the animal strains employed once a palpable tumour has been observed, i.e. when the inoculum has been successfully grafted. Whenever sufficient material was available the tests were done on at least two of the above types of tumour. The tumour passages and animal tests were carried out by two of the authors (D.H. and D.L.W.).

*General technique.*

Mice bearing small implanted tumours were injected subcutaneously or intraperitoneally with 0·2 to 0·5 ml. of solution or suspension of the chemical on alternate days and observed for regression or inhibition of growth. It is of course necessary to ensure that any effects observed on the tumour development are not due simply to toxic chemical factors affecting the health of the animal. In our experiments the dose given was usually appreciably less than the amount which produced perceptible impairment of health.

In a few cases injections were given the day following implantation of the tumours, with subsequent injections every other day. In all experiments the injected mice were compared with an equal number of control mice, approximately 10 to 12 being used for each test, which was continued for 10 to 14 days. The overall results were identical by either technique, and since these did not indicate any significant inhibition, the size and growth rates have not been detailed.

In some supplementary tests the compounds were injected intravenously in doses roughly two-fifths of those recorded below. When practicable, the compounds tested were injected in aqueous solution or, in the case of the acidic compounds, in the presence of a suitable buffer. When the substance was not sufficiently water-soluble, colloidal solutions were prepared, usually with the addition of a stabilizer, such as a small amount of gum or gelatine. Two compounds (cholyl cyanide and cholesteryl chloride, No. 6 and 15) were injected subcutaneously dissolved in cod liver oil.

*Materials.*

The syntheses of certain chemicals made specially for these studies are described in a later section. Most of them (No. 10, 14, 18, 22 to 29) were prepared by workers in the Department of Chemistry, University of Birmingham. The compounds (36 to 38) in Group D and all those in Group E were obtained from the Research Department of May & Baker, Ltd. (Dagenham, Essex), and made available through the co-operation of Dr. A. J. Ewins. Those in Group F were prepared in the Birmingham Medical School Cancer Research Laboratories.

### *Group A.*

*Compounds related to the sterols and bile acids.*

This series (Table I) was selected from substances prepared during researches on cholic acid and its derivatives, which are of particular interest in view of their bacteriostatic properties (Stacey and Webb, 1947; Jones, Smith and Webb, 1948). The *in vitro* antibacterial action of the bile acids, particularly against certain Gram-positive micro-organisms, is well known (Sobotka, 1937). It has also been observed that certain bile salts are able to remove the ribonucleic acid component and other vital constituents from the surface of certain types of bacteria (Henry and Stacey, 1943). Components structurally related to the bile acids were therefore submitted to *in vivo* tests on tumours to determine whether they would produce any effect on the growth of mammalian neoplastic cells.

For this study an endeavour was made to produce substances in which the original structure was altered in various ways, by the addition of new polar groups (acid or basic), or by substitution with non-polar radicals in various positions.

The compounds and dosage per injection are given in Table I. As indicated previously, in no case was any significant inhibition of tumour growth observed.

TABLE I.

| | Compound. | Dosage (mg.). |
|---|---|---|
| 1. | 23-amino-3.7.12-trihydroxynorcholane | 2·0 |
| 2. | 23-Guanido ,, ,, | 1·0 |
| 3. | 24-Guanido-3.7.12-trihydroxycholane | 2·0 |
| 4. | 24-Amino ,, ,, | 1·5 |
| 5. | Cholyl amidine hydrochloride | 1·4 |
| 6. | Cholyl cyanide | 0·8 |
| 7. | Cholyl amide | 2·5 |
| 8. | Triformyl cholyl amide | 2·0 |
| 9. | Triformyl cholic acid | 0·5 |
| 10. | Triformyl cholyl diazoketone | 0·6 |
| 11. | Diformyl deoxycholic acid | 1·3 |
| 12. | Diacetyl deoxycholic acid | 0·7 |
| 13. | 3.7-Diformyl-12-ketocholanic acid | 0·8 |
| 14. | Dimesyl methyl deoxycholate | 0·5 |
| 15. | Cholesteryl chloride | 1·5 |
| 16. | Cholyl hydrazine | 1·0 |
| 17. | Cholyl ethyl urethane | 0·1 |
| 18. | 23-N-methyl amino-3.7.12-trihydroxynorcholane | 1·0 |
| 19. | 3-amino-7.12-dihydroxycholanic acid | 0·3 |
| 20. | Isoamyl-3-amino-7.12-dihydroxycholanate | 0·5 |
| 21. | Methyl-2-amino-7.12-dihydroxycholanate | 0·5 |

The syntheses of the majority of the compounds listed in Table I have been recorded elsewhere, and require little further comment. The methods for the preparation for compounds 1 to 8 have been described by James, Smith, Stacey and Webb (1946), and for compounds 19 to 21 by Jones, Webb and Smith (1949). An improved method for the preparation of the formyl derivatives 8, 9, 11 and 13 has also been recorded (Hughes, Smith and Webb, 1949). References to the preparation of compounds 12, 15, 16 and 17 are summarized by Sobotka (1937).

### *Group B.*

*Amino compounds.*

A considerable amount of research has been carried out on the chemotherapeutic properties of aromatic amino derivatives of several types. Boyland (1946) found that a number of diamino-diphenyl compounds inhibited growth of spontaneous tumours in mice, while other amino compounds which have given some measure of success with mouse and rat tumours include the N-substituted amino stilbenes, and the related stilbamidines (Haddow, Harris, Kon and Roe, 1948). None of the compounds we tested (Table II) had any measurable effect on the rate of growth of the implanted tumours.

TABLE II.

| | Compound. | Dosage (mg.). |
|---|---|---|
| 22. | 1.2-naphthadiamine | 0·2 |
| 23. | Glucose-β-naphthanilide | 0·5 |
| 24. | Glucose-α-naphthanilide | 0·2 |
| 25. | N-acetyl-9-amino-anthracene | 0·4 |
| 26. | Diaminophenanthrene | 0·1 |
| 27. | Diaminophenenthraquinone | 1·2 |
| 28. | 6-aminochrysene | 0·4 |
| 29. | 6-12-diaminochrysene | 0·2 |

*Group C.*

*Halogeno compounds.*

Some chlorinated hydrocarbons have been found to have strong insecticidal activity, often believed to be associated with their solubility in lipoids. Also these and other chlorinated ethanes and ethylenes have been found to affect the germination of seeds, and in particular to cause the liberation of glutathione (Guthrie, 1939).

In view of the special attributes of fluorine in organic compounds, it was considered that a short series of halogeno, saturated and unsaturated hydrocarbons, including some containing fluorine, should be tested for their capacity to inhibit tumour growth (Table III).

TABLE III.

| | Compound. | Dosage (mg.). |
|---|---|---|
| 30. | Phenyl pentachloride | 5·0 |
| 31. | 2.2′-Diphenyl-1.1.1-trichloroethane | 0·1 |
| 32. | 2.2′-Di(*p*-fluorophenyl)-1.1.1-trichloroethane | 1·0 |
| 33. | 4.4′-Difluorobenzophenone | 0·8 |
| 34. | Tolan tetrachloride | 0·2 |

*Group D.*

*Compounds with redox characteristics.*

Strauss, Cheronis and Strauss (1948) reported that with malignant tissues reduced tetrazolium salts to the red " formazan " form much more rapidly than normal tissues. The group of compounds listed below (Table IV) are of this type, and it was anticipated that they might have some effect on, for example, the dehydrogenase systems of the tumours. However, no inhibition whatsoever was observed with the doses given. Moreover, we were unable to confirm the original observation of Strauss, Cheronis and Strauss (1948) by tests on tumour or normal tissue slices, and Seligman, Gofstein and Rutenburg (1949) have since reported that there is no evidence of localization of such compounds in animal tumours when $I^{131}$-tagged compounds are used.

TABLE IV.

| | Compound. | Dosage (mg.). |
|---|---|---|
| 35. | 2.3.5-Triphenyltetrazolium chloride . . . . | 0·1 |
| 36. | 3.5-diphenyl-2*p*-$NH_2$-phenyl-tetrazolium chloride | 0·1 |
| 37. | 2.3.diphenyl-5*p*-$NH_2$- ,, ,, ,, | 0·1 |
| 38. | 2.5.diphenyl-3*p*-NHAc- ,, ,, ,, | 0·1 |
| 39. | Resazurin . . . . . . . . | 3·0 |

35 and 39 were ordinary reagent quality chemicals.

*Group E.*

*Intermediates in the synthesis of penicillic acid.*

The series of derivatives in Table V are intermediates obtained during studies on the synthesis of penicillic acid which all possess the basic skeleton

They were tested because of the biological (bactericidal) activity of penicillic acid and related compounds.

TABLE V.

| | Compounds. | Dosage (mg.). |
|---|---|---|
| 40. | 5-Methyl-4-hydroxyhex-5-en-2-inoic acid . . . . . . | 0·8 |
| 41. | 5-Methyl-4-hydroxy-3-methoxyhexa-2:5 dienoic acid lactone . . | 1·0 |
| 42. | 5-Methyl-4-hydroxy-3-methoxyhexa-2:4 dienoic acid lactone . . | 0·4 |
| 43. | 6-Bromo-5-Methyl-4-hydroxy-3-methoxyhexa-2:4-dienoic acid lactone . . . . . . . . . . . | 0·4 |
| 44. | 5-Methyl-5-hydroxy-3-methoxy-4-ketohex-2 enoic acid . . . | 5·0 |

*Group F.*

*Zinc derivatives.*

Evidence has been found of significant differences between the zinc content of normal and some malignant tissues, the latter often having a much higher content (Heath, 1949).

Furthermore, various amino acids (Beard, 1942) and malonic acid have been found to have inhibitory effects on tumour growth (Boyland, 1940; Woodhouse, 1947). The inhibition in the form of such co-ordinated metallic compounds was thought to offer some advantage. The compounds showed moderate toxic properties ; the doses shown in Table VI are close to the maximum tolerated by animals of 25 to 30 g. weight given repeated injections.

TABLE VI.

| | Compounds. | % Zinc. | Dosage (mg.). |
|---|---|---|---|
| 45. | Zinc-glycine . . | 15·5 . | 1·0 |
| 46. | Zinc-tyrosine . . | 10·1 . | 1·3 |
| 47. | Zinc-glutamic acid . . | 20·4 . | 0·9 |
| 48. | Zinc-Malonic acid . . | 31·5 . | 1·0 |

The compounds were prepared by warming the amino acid in the presence of excess freshly precipitated zinc hydroxide. After filtering off excess zinc hydroxide the compounds were isolated by careful evaporation and crystallization. The zinc content was determined on the dried preparations by Lang's ferricyanide-iodide method.

### *Special Syntheses.*

*Triformyl cholyl diazoketone* (10), isolated as an intermediary in the synthesis of homocholic acid, was prepared from triformyl cholyl chloride and diazomethane according to the method of Ruzicka, Plattner and Heusser (1944). Repeated recrystallization from Grignard-dried methanol gave triformyl cholyl diazoketone as white needles (m.p. 135 to 136°, $[\alpha]_D^{20} + 86{\cdot}2$, in chloroform (c = 1·1). (Found: C, 64·9; H, 7·6.; Calc. for $C_{28}H_{40}O_7N_2$; C, 65·1; H, 7·7 per cent.)

*Dimesyl methyl deoxycholate* (14).—Methyl deoxycholate (2 g.) was thoroughly dried by distillation with sodium-dry ethanol and benzene, and the resulting syrup dissolved in dry pyridine (4 c.c.). The solution was cooled in ice, and freshly distilled methane sulphonyl chloride (1 c.c.) added in 5 equal proportions of 0·2 c.c., over a period of approximately 1 hour. After standing at room temperature for 3 days, a few drops of water were stirred into the dark nearly-solid mixture, and the whole stirred into excess water. The sticky solid which separated hardened on trituration, and was filtered off. The aqueous filtrate was extracted with chloroform (6 times) and the extract washed with water, ice-cold 1N hydrochloric acid (5 times) to remove pyridine, again with water, sodium bicarbonate solution, and finally with water. After drying ($CaCl_2$), evaporation of this extract yielded only a very small amount of a brown syrup, indicating that only a small fraction of the product remained in aqueous solution.

The filtered solid was dissolved in ethanol and a little acetone, and the solution slowly run into water (800 c.c.) with vigorous stirring. The brownish solid which separated was filtered off and dried. It could not be crystallized from any of the various solvents used. The product was therefore dried as before by distillation with dry benzene and ethanol, dissolved in dry pyridine and again treated with mesyl chloride (1 c.c.). After standing overnight at room temperature the solution was heated at 50° for 1 hour, and then worked up as before. An initial purification was effected by dissolving the crude product in acetone and stirring into brine. The precipitated solid was dissolved in acetone and the solution decolorized with charcoal. Evaporation of the solution gave a syrup which crystallized in colourless monoclinic plates from a mixture of dry acetone, dry benzene and dry light petroleum. The crystals (1·05 g., 38 per cent) had m.p. 63°, depressed to 57° in admixtures with methyl deoxycholate (m.p. 63·50), and showed $[\alpha]_D^{12} - 50°$ in chloroform (*c*, 1·0). (Found: S, 11·8; Calc. for $C_{27}H_{46}O_8S_2$; S, 11·4 per cent.)

23-*N-methylamino*-3:7:12-*trihydroxynorcholane* (18).—An attempt was made to increase the basicity of 23-amino-3:7:12-trihydroxynorcholane by the formation of its N-methyl derivative following the method of Eckhardt (1938) for the preparation of N-methyl-7-amino-cholestane. The first step in the reaction involved the preparation of the free base from the hydrochloride. Neutralization of an ethanolic solution of the latter with sodium hydroxide in ethanol proved unsuitable, since water liberated during the reaction, as well as traces of carbonate in the alkali, tended to react with the free base. The following method of synthesis proved suitable:

A solution of 23-amino-3:7:12-trihydroxynorcholane-hydrochloride (2 g.) in ethanol (10 ml.) containing sodium ethoxide (from 0·11 g. Na) was kept at 60° for 2 hours. The cooled solution was filtered to remove the precipitate of sodium chloride, and evaporated to dryness under diminished pressure at 50° (bath temperature) in an atmosphere of nitrogen. The dry residue was extracted (3 times) under reflux with dry ether (10 ml.). The combined ethereal extracts (in nitrogen) were cooled in ice and methyl iodide (3·5 ml.) added. The white crystalline product which separated on standing overnight at 0° was collected and dried *in vacuo* at room temperature. Recrystallization from acetone containing hydriodic acid (0·02N) gave N-methyl-23-amino-3:7:12-trihydroxynorcholane hydroiodide (0·8 g., m.p. 268 to 269°). Found: C, 54·9 ; H, 2 ; N, 2·4. $C_{24}H_{44}O_3NI$ requires C, 55·1 ; H, 8·4 ; N, 2·6 per cent.)

1:2 *Naphthadiamine* (22).—Treatment of 2-naphthylamine (10 g.) in warm ethanol (65 ml.) with a solution of benzene diazonium chloride from aniline (7 g.) according to the method of Bamberger and Schieffelin (1889) gave 1-Benz-azo-2-naphthylamine (10·2 g., m.p. 102–104°, after recrystallization from ethyl acetate). The latter compound (5 g.) was reduced with zinc dust and acetic acid in the usual way, and the resulting solution poured into 0·2 N sulphuric acid (100 ml.). The precipitated sulphate was dissolved in warm 5N sodium hydroxide (40 ml.) and the solution filtered. On cooling, light brown crystals separated, which were filtered, washed with water and dried. Recrystallization from aqueous ethanol yielded needles of 1:2-diaminonaphthalene (1·2 g., m.p. 97°).

*Glucose-β-naphthadiamine* (23).—A solution of 2-naphthylamine (2 g. purified by sublimation at 112°) in ethanol (30 ml.) was boiled under reflux with glucose (1 g.) for 1 hour. Excess ethanol was then removed by evaporation, and the residual solution treated with ether (80 to 100 ml.). The crystals of glucose-β-naphthanilide (0·8 g., m.p. 115 to 117°) which separated on standing were filtered, washed with ether and dried *in vacuo.*

*Glucose- -napththanilide* (24) was prepared from 1-naphthylamine (purified by sublimation at 50°) and glucose in an identical manner.

*N-acetyl-9-aminoanthracene* (25).—9-Aminoanthracene was prepared from anthracene by the formation and subsequent reduction of 9-nitroanthracene according to the method of Dimroth (1901). The N-acetyl derivative was formed when a solution of 9-aminoanthracene (1·2 g.) in redistilled acetic anhydride (7 ml.) was allowed to stand in the cold. The crystalline product which separated was collected, washed first with acetic acid and then with ethanol and dried. Recrystallization from aqueous ethanol yielded N-acetyl-9-aminoanthracene (0·8 g., m.p. 273 to 274°).

9:10-*Diaminophenanthrene* (26) was obtained by the reduction of phena-n thraquinone dioxine in ethanol solution with zinc chloride-hydrochloric acid according to the method of Schmidt and Soll (1907). The crude product was recrystallized from ethanol to give 9:10 diamino phenanthrene (1·1 g., m.p. 154 to 155°) as needles. (Found: N, 16·4 ; Calc. for $C_{14}H_{12}N_2$ ; N, 16·6 per cent.)

2:7-*Diaminophenanthraquinone* (27).—A solution of phenanthraquinone (10 g.) and methylamine hydrochloride (8·8 g.) in dry benzene (35 ml.) was boiled under reflux for 6 hours (Jaffe and Day, 1943). The residual solid was extracted repeatedly with boiling benzene (50 ml.). The combined benzene extracts were filtered to remove unchanged phenanthraquinone (2·1 g., m.p. 185 to 195°), and evaporated under reduced pressure at 50° (bath temperature). The residual yellow solid was extracted with 5N hydrochloric acid, ethanol, and then with ethylacetate. Evaporation of the ethyl acetate extract to half-volume under reduced pressure gave a solid which, on crystallization and recrystallization from ethanol, afforded 2:7-diaminophenanthraquinone (1·2 g., m.p. 209 to 210°).

6-*Amino-chrysene* (28) was prepared by the following modification of the method of Newman and Cathcarte (1940). Reduction of the intermediary, 6-nitro-chrysene was more efficiently carried out with iron filings and hydrochloric acid than with red phosphorus and hydriodic acid.

To a solution of chrysene (10 g.) in glacial acetic acid (280 ml.) was added a mixture of concentrated nitric acid (8 ml.) and concentrated sulphuric acid (10 ml.) in glacial acetic acid (100 ml.). The yellow precipitate which separated from the reaction mixture was collected after 30 minutes, dried and recrystallized from aqueous pyridine. The resulting product was dissolved in absolute benzene, and the solution passed first through a column of alumina and then through silicon dioxide. Evaporation of the benzene solution gave 6-nitro chrysene (7·5 g, m.p. 212 to 213°).

*Reduction of* 6-*nitrochrysene.*

(a) *With red phosphorus and hydriodic acid.*—A mixture of 6-nitrochrysene (2 g.) and red phosphorus (0·5 g.) in hydriodic acid (D 1·7, 4·5 ml.) was heated under reflux on a sand tray and glacial acetic acid (10 ml.) cautiously added. After 1 hour the mixture was cooled, and the reddish-brown solid which separated extracted several times with benzene (60 ml.). The combined benzene extracts were washed with water, sodium thiosulphate solution and again with water. After drying ($MgSO_4$), the solution was concentrated under diminished pressure in an atmosphere of nitrogen at 40° (bath temperature) to yield a white solid (m.p. 270 to 276°). The latter was rapidly filtered and dissolved immediately in acetic anhydride (8 ml.) and glacial acetic acid (3 ml.). After 30 minutes at room temperature the solution was poured into water, and the white precipitate which separated collected, washed with water and dried. Recrystallization from aqueous ethanol afforded 6-N-acetyl-aminochrysene (0·37 g., m.p. 297 to 299°).

(b) *Iron filings and hydrochloric acid.*—6-nitrochrysene (4 g.) was added with vigorous stirring to a mixture of iron filings (2·1g.), 5 N-hydrochloric acid (20 ml.), and water (5 ml.) at 95 to 100°; when the 6-nitrochrysene had dissolved completely the heating was discontinued, and sodium carbonate (6 g.) added. The solution was then cooled, filtered and evaporated under reduced pressure. The residue was extracted with ethanol, the combined extracts dried and evaporated under reduced pressure at 40°. The crystalline product which separated was recrystallized from ethanol to give 6-aminochrysene (2·7 g., m.p. 274 to 275°).

6:12-*Diaminochrysene* (29).—Chrysene (6 g.) in glacial acetic acid (210 ml.) was treated at 90° with concentrated nitric acid (63 ml.) and concentrated sulphuric acid (6 ml.) to give 6:12-dinitrochrysene (7·3 g., m.p. 372 to 376°). The latter (5 g.) was added slowly with vigorous stirring to a mixture of 5*N* hydrochloric acid (25 ml.) and iron filings (4·2 g.) at 95°. After 2 hours sodium carbonate (5 g.) was added, the solution cooled and filtered. The filtrate was extracted several times with ether (100 ml.), the combined ethereal extracts washed with water, dried ($MgSO_4$), and evaporated to dryness. The residual white solid, on recrystallization from ethanol, gave 6:12 diaminochrysene (3·9 g.), which was converted to its stable diacetyl derivative in the usual way. Recrystallized from aqueous ethanol, the 6:12-diacetylamino chrysene formed needles (362 to 365°).

SUMMARY.

Forty-eight chemicals belonging to 6 general groups have been tested for tumour inhibition by injection into mice bearing grafted sarcomas and carcinomas with negative results.

The groupings were :

A. Compounds related to the sterols and bile acids.
B. Amino compounds.
C. Halogeno-compounds.
D. Compounds with Redox characteristics.
E. Intermediates in the synthesis of penicillic acid.
F. Zinc derivatives of amino acids.

The syntheses of the compounds specially prepared for this work are described.

This work was carried out on behalf of the Birmingham Branch of the British Empire Cancer Campaign.

REFERENCES.

Bamberger, F., and Schieffelin, W.—(1889) *Ber. dtsch. chem. Ges.*, **22**, 1374.

Beard, H. H.—(1942) *Arch. Biochem.*, **1**, 177.

Boyland, E.—(1940) *Biochem. J.*, **34**, 1196.—(1946) *Ibid.*, **40**, 55.

Dimroth, O.—(1901) *Ber. dtsch. chem. Ges.*, **34**, 219.

Eckhardt, H. J.—(1938) *Ibid.*, **71**, 461.

Guthrie, J. M.—(1939) *Contr. Boyce Thomson Inst.*, **10**, 325.

Haddow, A., Harris, R. J. C., Kon, G. A. R., and Roe, E. M. F.—(1948) *Philos. Trans.*, A, **241**, 147.

Heath, H. C.—(1949) *Nature*, **164**, 1055.

Henry, H., and Stacey, M.—(1943) *Ibid.*, **157**, 671.

Hughes, I. W., Smith, F., and Webb, M.—(1949) *J. chem. Soc.*, p. 3437.

Jaffe, G. M., and Day, A. R.—(1943) *J. org. Chem.*, **8**, 43.

James, S. P., Smith, F., Stacey, M., and Webb, M.—(1946) *J. chem. Soc.*, p. 665.

Jones, A. S., Smith, F., and Webb, M.—(1948) *Nature*, **162**, 857.

*Idem*, Webb, M., and Smith, F.—(1949) *J. chem. Soc.*, p. 2764.

McDonald, S., Jr., and Woodhouse, D. L.—(1938) *J. Path. Bact.*, **47**, 615.

Newman, M. S., and Cathcarte, J. A.—(1940) *J. org. Chem.*, **5**, 618.

Ruzicka, L., Plattner, A., and Heusser, H.—(1944) *Helv. chim. Acta*, **27**, 186.

Schmidt, J., and Soll, J.—(1907) *Ber. dtsch. chem. Ges.*, **40**, 2454.

Seligman, A. M., Gofstein, R., and Rutenburg, A. M.—(1949) *Cancer Res.*, **9**, 366.

Sobotka, H.—(1937) ' Physiological Chemistry of the Bile.' London (Ballière, Tindall & Cox), p. 125.

Stacey, M., and Webb, M.—(1947) *Proc. Roy. Soc.*, **134**, 523.

Strauss, F. H., Cheronis, N. D., and Strauss, E.—(1948) *Science*, **108**, 113.

Woodhouse, D. L.—(1947) *Cancer Res.*, **7**, 398.